# Revisiting hydroxychloroquine and chloroquine for patients with chronic immunity-mediated inflammatory rheumatic diseases

**DOI:** 10.1186/s42358-020-00134-8

**Published:** 2020-06-09

**Authors:** Edgard Torres dos Reis Neto, Adriana Maria Kakehasi, Marcelo de Medeiros Pinheiro, Gilda Aparecida Ferreira, Cláudia Diniz Lopes Marques, Licia Maria Henrique da Mota, Eduardo dos Santos Paiva, Gecilmara Cristina Salviato Pileggi, Emília Inoue Sato, Ana Paula Monteiro Gomides Reis, Ricardo Machado Xavier, José Roberto Provenza

**Affiliations:** 1 Disciplina de Reumatologia, Escola Paulista de Medicina, Universidade Federal de São Paulo, São Paulo, Brazil; 2 Serviço de Reumatologia do Hospital das Clínicas da Universidade Federal de Minas Gerais, Belo Horizonte, Brazil; 3 Serviço de Reumatologia do Hospital das Clínicas da Universidade Federal de Pernambuco, Recife, Brazil; 4 Hospital Universitário - UnB/EBSERH, Brasília, Brazil; 5 Serviço de Reumatologia do Hospital das Clínicas da Universidade Federal do Paraná, Curitiba, Brazil; 6 Instituto de Ensino e Pesquisa (IEP), Hospital Amor, Barretos, Brazil; 7 Serviço de Reumatologia do Hospital de Clínicas de Porto Alegre da Universidade Federal do Rio Grande do Sul, Porto Alegre, Brazil; 8 Pontifícia Universidade Católica de Campinas, Campinas, Brazil

**Keywords:** Hydroxychloroquine, Chloroquine, Antimalarials, Chronic immune-mediated inflammatory rheumatic diseases

## Abstract

Hydroxychloroquine and chloroquine, also known as antimalarial drugs, are widely used in the treatment of rheumatic diseases and have recently become the focus of attention because of the ongoing COVID-19 pandemic. Rheumatologists have been using antimalarials to manage patients with chronic immune-mediated inflammatory rheumatic diseases for decades. It is an appropriate time to review their immunomodulatory and anti-inflammatory mechanisms impact on disease activity and survival of systemic lupus erythematosus patient, including antiplatelet effect, metabolic and lipid benefits. We also discuss possible adverse effects, adding a practical and comprehensive approach to monitoring rheumatic patients during treatment with these drugs.

## Background

Hydroxychloroquine (HCQ) and chloroquine (CQ), known as antimalarial (AM) drugs, are widely used in the treatment of rheumatic disorders, especially in immune-mediated such as systemic lupus erythematosus (SLE), cutaneous lupus [1–4] and rheumatoid arthritis (RA) [5, 6]. Besides that, both the Brazilian Society of Rheumatology (SBR) and the European League Against Rheumatism (EULAR) recommend, in specific circumstances, the use of HCQ for primary Sjögren syndrome (pSS) [7, 8] and antiphospholipid syndrome (APS) [9]. HCQ is currently preferred over chloroquine as it has a better safety profile [10], especially regarding the risk of retinopathy [11].

In this narrative review, the mechanism of action of these medications, as well as their main clinical, biological and safety effects in patients with chronic immune-mediated inflammatory rheumatic diseases (CIMID) will be discussed. Therefore, studies of these drugs related to COVID-19 will not be addressed in this review.

## Methods

The new scenario of COVID-19 pandemic brought many medical challenges to physicians and health care systems. In view of this situation, The Brazilian Society of Rheumatology established a team of specialists from its commissions to respond to the demands related to the topic, especially those come from the Brazilian Ministry of Health. The discussion about the possible use of AM in SARS-cov2 infection showed the opportunity to revisit the topic by rheumatologists. A writing committee started gathering published research and analyzed it carefully. After discussion and debate, the committee members agreed on what would be the most useful knowledge to be highlighted about AM for rheumatologists, and prepared this manuscript. In a time of rapid response to a public health emergency, this type of document needed to be produced quickly and was evidence-informed, but not supported by complete evidence reviews.

## Pharmacological characteristics

**Fig. 1:**
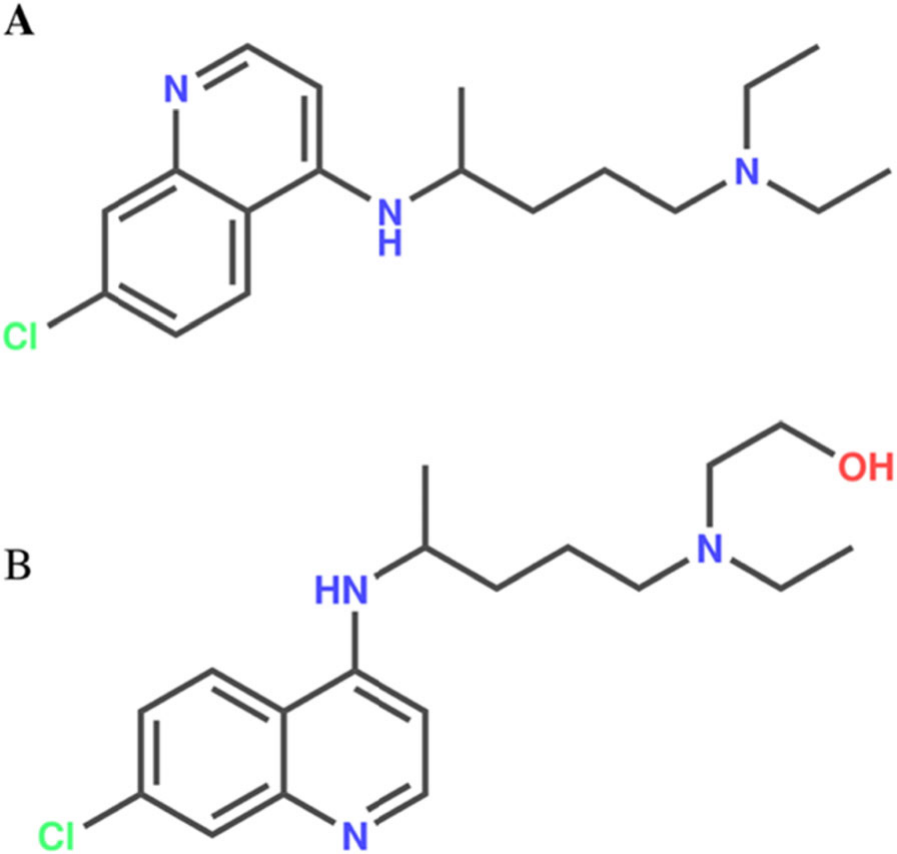
Chemical structure of chloroquine (**a**) and hydroxychloroquine (**b**)

CQ is a 4-aminoquinoline known since 1934, discovered in the first half of the twentieth century as an effective substitute for quinine. Currently, CQ is the drug of choice for the treatment of malaria [12]. Hydroxychloroquine is a hydroxylated analogue of CQ that has both antimalarial and antiinflammatory activities (Fig. 1). These two molecules enter cells as non-protonated forms and become protonated, inversely proportional to pH, according to Henderson-Hasselbach’s law. Therefore, these drugs are concentrated in acidic organelles, including endosomes, lysosomes and Golgi vesicles, increasing the pH [13].

Both drugs are weak bases and have a large volume of distribution with a half-life of about 50 days. These drugs interfere with lysosomal activity and autophagy, interact with membrane stability and may alter signaling pathways and transcriptional activity, resulting in inhibition of cytokine production and modulation of certain co-stimulatory molecules. At the cellular level, they inhibit the Toll-like receptors signaling and reduce the CD154 molecule expression in T cells. Effects on plasmacytoid dendritic cells (pDCs), B cells and other antigen presenting cells have also been described [13].

HCQ is administered as a sulfate while chloroquine is administered as a phosphate salt. The differences between the pharmacokinetic properties of CQ and HCQ are presented in Table 1.

**Table 1:** Pharmacokinetic properties of chloroquine and hydroxychloroquine

	Chloroquine	Hydroxychloroquine
Oral absorption	Upper gastrointestinal tract	Upper gastrointestinal tract
Distribution volume	Blood 65,000 L Plasma 15,000 L	Blood 47,257 L Plasma 5500 L
Hepatic metabolism	Desethylchloroquine 39%	Desethylchloroquine 18% Desethyl-hydroxychloroquine 16%
Renal clearance	51%	21%
Unmetabolized excretion	58%	62%
Terminal half-life	41 ± 11 days	45 ± 15 days

Adapted from: Schrezenmeier E et al. [13]

## Mechanisms of action

**Fig. 2:**
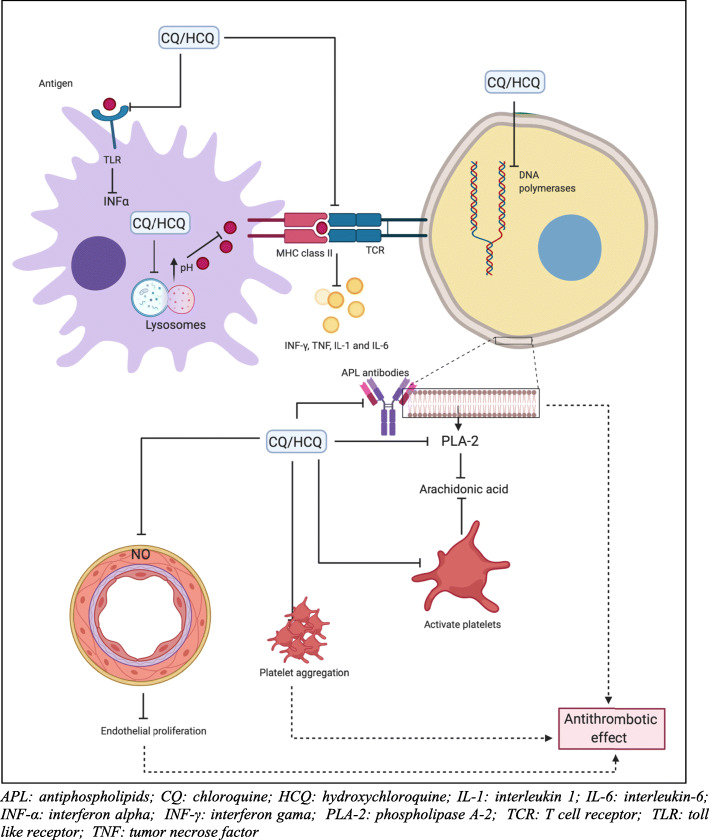
Proposed mechanisms of action of antimalarials (chloroquine and hydroxychloroquine). APL: antiphospholipids; CQ: chloroquine; HCQ: hydroxychloroquine; IL-1: interleukin 1; IL-6: interleukin-6; INF-α: interferon alpha; INF-γ: interferon gama; PLA-2: phospholipase A-2; TCR: T cell receptor; TLR: toll like receptor; TNF: tumor necrose factor

The exact mechanism of action of HCQ and CQ in the treatment of CIMID is not yet fully understood, but there is strong evidence that they have an immunomodulatory and antithrombotic effect [13, 14]. The proposed mechanisms to explain these effects are (Fig. 2):

- Alkalinization of lysosomes and other intracellular acid compartments with interference in phagocytosis. The increase of intracellular pH causes a selective change in the presentation of proper antigens;
- Blockage of T-cell response and reduction of pro-inflammatory cytokine production, including INF-γ, TNF, IL-1 and IL-6;
- Blockage of Toll-like receptors 7 and 9, especially in plasmacytoid dendritic cell with inhibition of INF-α, which plays an important role in the pathophysiology of SLE;
- cGAS-STING signaling inhibition;
- Inhibition of phospholipase A2 activity;
- Stimulation of nitric oxide production by endothelial cells with antiproliferative effect;
- Antithrombotic effect through the inhibition of platelet aggregation in a dose-dependent manner, decreased production of arachidonic acid by activated platelets and action on antiphospholipid antibodies.
- Action on glucose metabolism and lipid profile as a non-immunomodulatory mechanism

## Benefits in SLE

This class of medication has been used in the treatment of SLE for more than 50 years. It is a chronic autoimmune inflammatory disease that can affect several organs and systems and has a variable incidence, with 8.7 cases/100,000 inhabitants in Brazil [15]. It mainly affects young women aged from 15 to 45 years-old with heterogeneous and pleomorphic clinical manifestations [16, 17].

In 1976, Urowitz et al. described a bimodal mortality pattern in lupus patients, with premature deaths related to disease activity or infection, while late rate was more associated with atherosclerotic disease [18]. Considering the improvements in diagnosis and treatment, as well as the reduction of complications related to disease itself or its own treatment, the survival rate has increased in the two last decades [19].

The treatment of SLE patients may be individualized and targeted, according to the disease activity and severity. Additionally, patient education about the disease, sun exposure protection, regular physical exercise, diet, treatment of comorbidities (hypertension, diabetes, dyslipidemia, osteoporosis), avoiding smoking and performing adequate contraception and vaccines are important approaches and should be stimulated for all patients. The main goals of treatment in SLE are to increase long-term survival, to induce and maintain remission, to prevent damage and to improve quality of life [10].

CQ or preferably HCQ should be always recommended for lupus patients, regardless other immunosuppressive medications and severity or type of clinical manifestations, except if some contraindication or previous toxicity [10, 11]. Both of them promote multiple benefits, including direct or indirect effects [20], such as reducing disease activity and new flares [21]; improvement of skin lesions and joint symptoms [22, 23]; prevention of accrual damage [24, 25]; possible mortality risk reduction [26, 27]; as well as some benefits on glucose and lipid metabolism and reduction of thrombotic phenomena [14].

### Disease activity

AMs are widely used and recommended for the treatment of SLE, since they promote an immunomodulatory effect of the immune response and better control of disease activity [2].

Tsakonas et al. demonstrated 57%-risk reduction of severe activity in quiescent lupus patients after HCQ, suggesting some prevention benefit on disease activity (RR = 0.43; 95% CI 0.17–1.12) [21]. The Canadian Hydroxychloroquine Study Group randomized 47 lupus patients, who were with stable dose of HCQ, to maintain on (n = 25) or to switch to placebo (n = 22). After 6-month HCQ withdrawal, there was significant 2.5-increase of SLE activity (95% CI 1.08–5.58; *p* = 0.02). Interestingly, there was a non-significant higher risk for severe activity, including vasculitis, transverse myelitis and nephritis, in those that had stopped the medication (RR = 6.1; 95% CI 0.72–52.54) [28]. Additionaly, some other studies have shown clinical worsening after drug discontinuation [29].

Regarding lupus nephritis, Petri et al. have demonstrated higher remission rate in 450 patients using HCQ when compared to non-users after 1-year follow-up (64% vs. 22%; *p* = 0.036) [30]. Also, the HCQ was a stronger predictor of complete renal remission in lupus patients when combined to mycophenolate than mycophenolate in monotherapy [31]. Thus, the SBR, the ACR, and the EULAR consensus and recommendations for treating lupus patients have recommended the HCQ as an adjunctive treatment [1–4].

### Damage accrual

Several studies have found relevant damage accrual in SLE patients [24, 32–35]. Accordingly to the LUMINA study, HCQ users had lower risk of developing new damage in patients with less than 5 years of disease (HR = 0.73; CI 95% 0.52–1.00; *p* = 0.05), especially in patients with no damage at baseline (HR = 0.55; CI 95% 0.34–0.87; *p* = 0.011) [24]. Another LUMINA analysis in 203 patients with lupus nephritis without renal damage found that HCQ delayed the onset of kidney failure (HR = 0.12; CI 95% 0.02–0.97; *p* = 0.046). The accumulated kidney damage was higher in HCQ non-users in class IV lupus nephritis [35]. Petri et al. evaluated 2054 patients and found that age, hypertension and use of corticosteroids were main predictors of damage, while HCQ had a protective effect (*p* = 0.06) [34].

### Thrombotic events

HCQ reduces platelet aggregation and its antithrombotic effect can be explained by the reduction of the formation of antiphospholipid-β2-glycoprotein complexes on monocytes surfaces [36] with protective effect in patients with SLE [26, 37–40] (OR = 0.17; CI 95% 0.07–0.44; *p* < 0.0001) [38] and HR = 0.28; CI95% 0.08–0.90) [26]. Jung et al. compared 54 patients with SLE and prior thrombosis with 108 lupus patients with no thrombosis and demonstrated that AM were associated with lower risk of thrombotic events, both arterial and venous (OR = 0.31; CI 95% 0.13–0.71) [41].

### Glucose metabolism and lipid profile

In vitro and experimental models demonstrated that HCQ improves insulin secretion and peripheral insulin sensitivity [14]. Penn et al. found HCQ was associated with lower fasting glycemia and Homeostatic Model Assessment (HOMA) index in patients with SLE [42].

Moreover, AM in monotherapy or associated with glucocorticoids (GC) have also improved the lipid profile in lupus patients because they provide hepatic cholesterol synthesis reduction with inhibition of lysosomal function, as well as lysosomal cholesterol transport and metabolism blockage. Other explanations are related to lower LDL receptor activity and bile steroid precursors and HMG-CoA reductase function gain [14]. Besides that, chloroquine diphosphate increases low-density lipoprotein removal from plasma in SLE patients [43].

Petri et al. found that HCQ was associated with lower total cholesterol serum levels, regardless dosage (200 mg or 400 mg/day), and it was able to mitigate the deleterious prednisone effect (10 mg/day) on total cholesterol [44]. Rahman et al. reported 4.1%-reduction of total cholesterol serum levels after starting AM in 3 months (*p* = 0.02). In 181 patients using GC and AM, the mean total cholesterol was 11% lower than in 201 patients receiving comparable dosage of GC (p = 0.002) [45]. Cairoli et al. demonstrated a significant decrease in total cholesterol (198 ± 33.7 vs. 183 ± 30.3 mg/dL; *p* = 0.023) and LDL levels (117 ± 31.3 vs. 101 ± 26.2 mg/dL; *p* = 0.023) after the 3 months of HCQ therapy in SLE patients which determined a significant decrease in the frequency of dyslipidemia (26% vs. 12.5%; *p* = 0.013) [46].

A recent systematic review and meta-analysis involving data from nine studies and 823 participants has stated that HCQ significantly reduced mean total cholesterol plasmatic levels (26.8 mg/dL; 95% CI 8.3–45.3), as well as mean LDL serum levels (24.3 mg/dL; CI95% 8.9–39.8). However, it is important to note that other studies had an extensive heterogeneity among them, including lack of information about statin use [47]. Similarly, there are controversial data regarding HDL status [14].

### Survival

Ruiz-Irastorza et al. evaluated a cohort with 232 lupus patients (64% on AMs). Among 23 patients who died, 19 (83%) had never received AMs. The cumulative 15-year survival rate was higher in those using AM drugs (0.98 vs. 0.15; *p* < 0.001) [26]. Shinjo et al., analyzing 1480 patients from the GLADEL (Grupo Latino Americano para Estudo do Lupus) found lower mortality rate in AMs users for at least six consecutive months (4.4% vs. 11.5%; *p* < 0.001). In addition, the protective effect on mortality rate increased according to longer exposition time to AMs [6 to 11 months: 3.85 (95%CI 1.41 to 8.37); 12 to 24 months: 2.7 (95%CI 1.41 to 4.76); and more than 24 months: 0.54 (95%CI 0.37 to 0.77)]. After adjustment to potential confounders, AMs were associated with a 38% reduction in mortality (HR = 0.62; 95%CI 0.39–0.99) [27].

### Pregnancy and lactation

The use of CQ and HCQ is not only allowed but is recommended during pregnancy and lactation in SLE patients [14]. A HCQ placebo-controlled study suggested beneficial effect on disease activity [48] and the interruption of HCQ was related to higher risk of flares during pregnancy. In other words, AMs are recommended during the preconception period, pregnancy and lactation [49].

The presence of anti-Ro/SSA and anti-La/SSB antibodies are associated with 1 to 2% risk of congenital total atrioventricular block. When there is a maternal history of an affected fetus or child, the recurrence rate can increase 13 to 18%. HCQ is associated with lower occurrence of neonatal cardiac lupus, especially if recurrent [50, 51].

More recently, a systematic review and meta-analysis involving 6 studies and 870 pregnancies have found no difference concerning prematurity and restricted intrauterine growth in lupus patients exposed (n = 308) or not exposed (n = 562) to HCQ. It is important to emphasize that these results should be addressed with caution due to huge heterogeneity among the studies [52].

## Benefits in RA

AMs are important as adjunctive therapy to treatment with disease-modifying drugs (DMARDs) in RA, including recommendations for treatment of SBR [5] and ACR [6]. HCQ has been shown to improve clinical and laboratory findings in RA, particularly in early and mild disease, although there was no protective effect on radiographic progression [53]. Because of its good safety profile, it currently being studied for the prevention of future onset of rheumatoid arthritis (RA) in individuals who have elevations of anti-cyclic citrullinated peptide (anti-CCP3) antibodies [54].

Similarly to data from lupus patients, most of effects are also seen in RA, including improvement in lipid profile and insulin resistance [55, 56]. In a multicenter study with 4905 RA patients, Wasko et al. demonstrated that HCQ was associated with lower risk of diabetes mellitus (HR = 0.62; 95% CI 0.42–0.92) [56] and could be used for controlling traditional cardiovascular risk factors [57].

## Benefits in other immune-mediated diseases

Antimalarial drugs may be used to treat sarcoidosis, including cutaneous sarcoidosis, pulmonary sarcoidosis, neurosarcoidosis, and arthritis [58]. Although less effective than in patients with SLE, AM can be useful in for cutaneous manifestations in dermatomyositis [59].

## Safety

AMs are usually effective, safe, and well tolerated. According to the SBR, the Brazilian Society of Dermatology and The Study Group on Inflammatory Bowel Diseases, patients with CIMID on AMs are considered as non-immunosuppressant medications [60]. There is no increased risk of infections or even neoplasms in the short- and long-term [61]. More frequently the adverse events are related to gastrointestinal complaints, such as abdominal pain, nausea, vomiting and diarrhea. To decrease these adverse effects, the HCQ can be taken once or twice daily with a meal [62].

Patients with psoriasis, porphyria and alcoholism may be more susceptible to adverse skin events, usually without severity. In rare cases, hemolysis may occur in patients with glucose-6-phosphate dehydrogenase deficiency [63]. Besides G6PD deficiency, the concomitant use of HCQ with dapsone may enhance the risk of hemolytic reactions [64].

There is no current recommendation to reduce the dose of HCQ in patients with chronic kidney disease [13]. Some experts recommend reducing the dose of chloroquine phosphate by 50% if the glomerular filtration rate is less than 10 mL/minute, and in hemodialysis or peritoneal dialysis patients [65]. Neither safety nor efficacy of HCQ has been established for chronic use in children for juvenile idiopathic arthritis or for juvenile SLE.

**Fig. 3:**
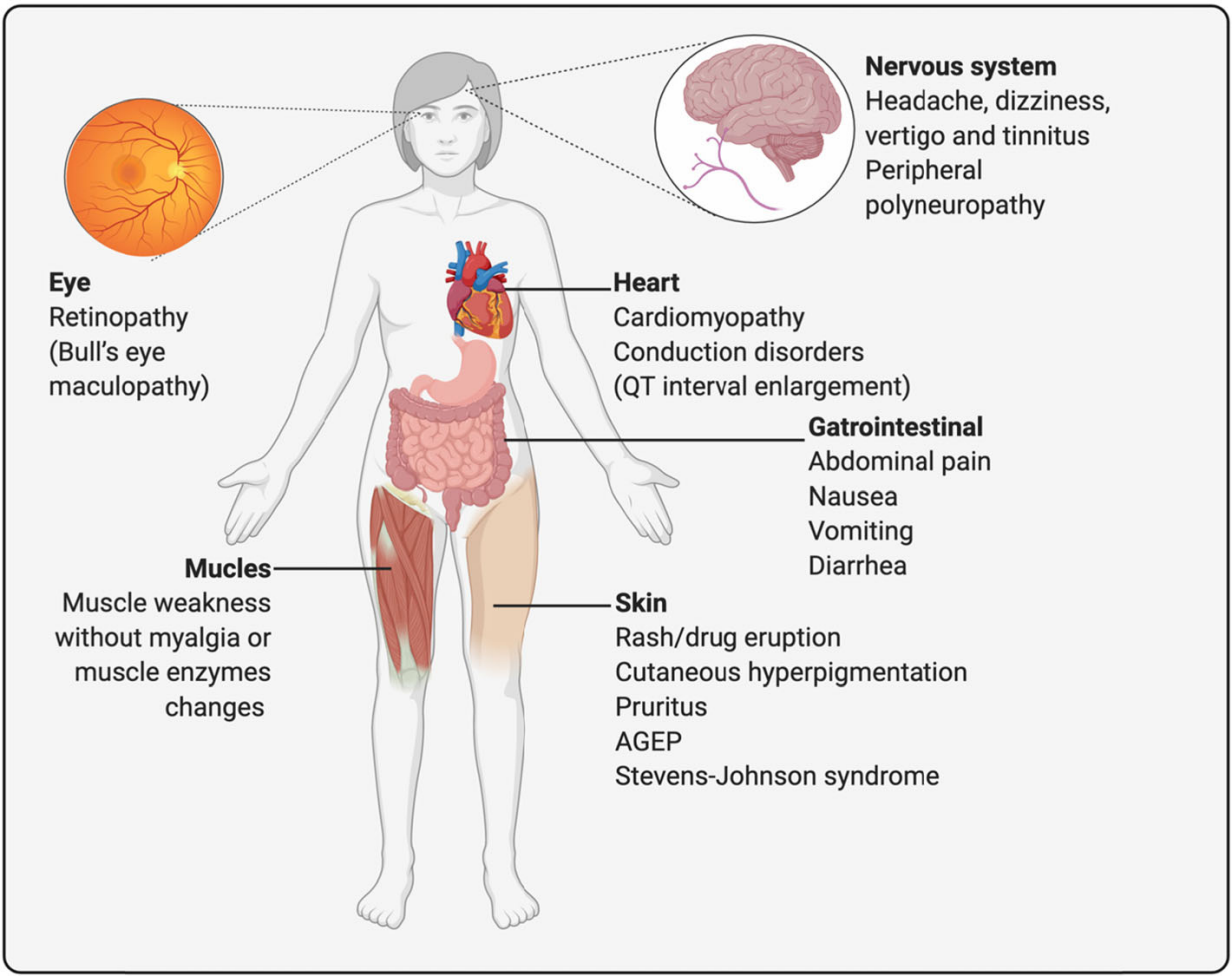
Main adverse events related to the use of antimalarials

The main adverse events related to the use of AM are summarized in Fig. 3.

### Ocular toxicity

Both CQ and HCQ can cause ocular deposition, an effect more associated with CQ. Retinal changes are related to lysosomal degradation of the external photoreceptor with lipofuscin accumulation in retinal pigment epithelium [66].

Once symptomatic, the retinopathy associated with AM is characterized by abnormalities of the retinal pigment epithelium, which are detectable clinically, and may later develop into the classic appearance of ‘bull’s eye maculopathy’ with retinal pigment epithelial loss. At this stage the visual loss is severe and irreversible and may be complicated by secondary cystoid macular oedema, epiretinal membrane and other sequalae [67].

Although rare, the retinopathy is one of main adverse events related to AMs [14]. Considering the recommended dosages, the 5-year, 10-year and 20-year toxicity risk is lesser than 1%, below 2 and 20%, respectively. After 20 years of use, the risk increases 4% each year for those no previous toxicity [11].

More recently, the hydroxychloroquinemia has been reported as a risk factor for retinopathy in 537 lupus patients (total prevalence = 4.3%) [68]. Other risk factors associated with retinopathy were age, duration of use and high body mass index (BMI).

In 2016, the American Society of Ophthalmology updated its recommendations for retinopathy screening in CQ or HCQ users. According to them, the maximum daily dosage of HCQ should be ≤5 mg/kg. The main risk factors for ocular toxicity are daily dose above the recommended, duration of use, renal failure, previous maculopathy or retinopathy and concomitant use of tamoxifen. Other risk factors include advanced age, liver failure and genetic factors related to abnormalities of the ABCA4 gene or cytochrome P450. It is recommended that patients initiating the drug undergo eye examination within the first year of treatment. Although visual field examination and spectral-domain optical coherence tomography (SD OCT) are very useful, they are not mandatory at the beginning of treatment, unless the patient has risk factors or other diseases that may affect the initial screening tests. In the absence of major risk factors, screening tests may be performed annually after 5 years of baseline assessment. If risk factors are present, screening tests should be performed annually or at shorter intervals soon after beginning AMs, and automated visual field assessment and OCT-SD are recommended. Additional tests in some situations may be indicated, such as the multifocal electroretinogram (mfERG), which provides objective information of visual field, especially in Asian patients [11].

### Adverse dermatologic events

The use of antimalarials may provoke adverse dermatologic effects of varying severity, being drug eruptions or rashes the most common [69]. Both CQ and HCQ bound to melanin and can deposit on the skin, with the possibility of cutaneous hyperpigmentation (grayish color) in long-term, especially with CQ [66].

A study that compared acitretin with HCQ for the treatment of cutaneous lupus found around 27% of patients with dry skin complaints; itching and burning sensation on the skin in 17%; dermatitis in 3% and desquamation in 3% of those using HCQ [70]. Also, grayish pigmentation of the skin and oral mucosa has been associated with longer use, higher levels of hydroxychloroquinemia, as well as the use of acetyl salicylic acid and oral anticoagulants, sometimes with reports of microtrauma and local ecchymosis preceding hyperpigmentation [71, 72]. Cases of worsening psoriasis are also described with the use of medication [73]. Acute generalized exanthematous pustulosis is rare and described in 1/5,000,000 inhabitants [74].

A recent systematic review including ninety-four articles, comprising a total of 689 adverse dermatologic side effects, has shown that drug eruption or rash (358 cases) were the most frequent, followed by cutaneous hyperpigmentation (116 cases), pruritis (62 cases), acute generalized exanthematous pustulosis (27cases), Stevens-Johnson syndrome or toxic epidermal necrolysis (26 cases), hair loss (12 cases), and stomatitis (11 cases) [69].

### Cardiotoxicity

Although rare, it can be a serious adverse event [75]. Both cardiomyopathy and conduction disorders (for example, QT prolongation) are described. A possible mechanism involves a lysosomal pathway dysfunction with metabolite products (glycogen and phospholipids) intracellular accumulation [76].

A systematic review about CQ and HCQ cardiotoxicity found 86 articles, comprising only 127 patients in case reports or small case series, most of them were SLE (n = 49) or RA patients (n = 28). Most patients (58.3%) were treated with CQ with a median time of use of 7 years (3 days to 35 years) and median cumulative dose of 803 g (1235 g for HCQ). Heart rhythm problems were the main reported side effects, affecting 85% of patients. Other non-specific cardiac events included ventricular hypertrophy, hypokinesia, valve dysfunction and pulmonary arterial hypertension. It is worth mentioning that 38 cases were classified as probably related to adverse drug events, 69 as possibly associated and in 20 cases it was not possible to indicate this association. It was not possible to classify this association as definitive for any case, using the Naranjo Scale. The authors could not definitively exclude the possibility that some cardiac complications were due to the disease itself or to differential diagnoses (Fabry disease, for example). Determination of the risk for cardiac complications attributed to the medications was not possible because of the lack of randomized controlled trials [75].

Other studies suggest that older age, duration of medication use, dosage above that recommended by weight, use of CQ instead of HCQ, pre-existing heart disease and renal failure may be risk factors for medication cardiotoxicity. In addition, the risk may be greater in those who use other medications that also lead to prolongation of the QT interval or that increase the serum level of QC [77–79]. A study suggested that SLE patients using AM drugs with persistently elevated creatine phosphokinase (CPK) should be monitored periodically and specific biomarkers, such as troponin and brain natriuretic peptide (BNP), may be useful as a screening tool for cardiotoxicity diagnosis by AMs. The electrocardiogram, echocardiogram and magnetic resonance imaging can provide more information in suspicious cases, as well as endomyocardial biopsy, if necessary [80]. At the moment, there are no consensus and guidelines which are the best methods and interval to monitor cardiotoxicity with chronic use of AM.

On the other hand, it is important to highlight HCQ and CQ have a protective effect on cardiovascular risk, anti-thrombotic mechanisms and on survival rate in lupus patients.

### Myotoxicity

It has been described in a few cases, especially associated to CQ. Patients with myopathy have proximal muscle weakness without myalgia or muscle enzymes changes (or slightly elevated more rarely). Patients can improve with medication discontinuation [63].

### Neurotoxicity

Central nervous system toxicity includes headache, dizziness, vertigo and tinnitus. There are rare case reports of seizures related to reduction of seizure threshold and psychosis, especially when combined to GC. Neuromyopathy and peripheral polyneuropathy are also rare, occurring in patients with worsening renal function and using CQ [62, 63].

### Drug interactions

HCQ and CQ are substrates for cytochrome P450 enzymes, responsible for the metabolism of multiple drugs. Cytochrome P450 enzymes dealkylate AMs to their active metabolites. Thus, the concomitant use of AMs can lead to increased levels of digoxin, cyclosporine and metoprolol [62]. HCQ can reduce gastrointestinal absorption of methotrexate, since it alters the local pH and it can explain lower toxicity of methotrexate when combined. Antacids may decrease oral bioavailability of CQ [13].

**Fig. 4:**
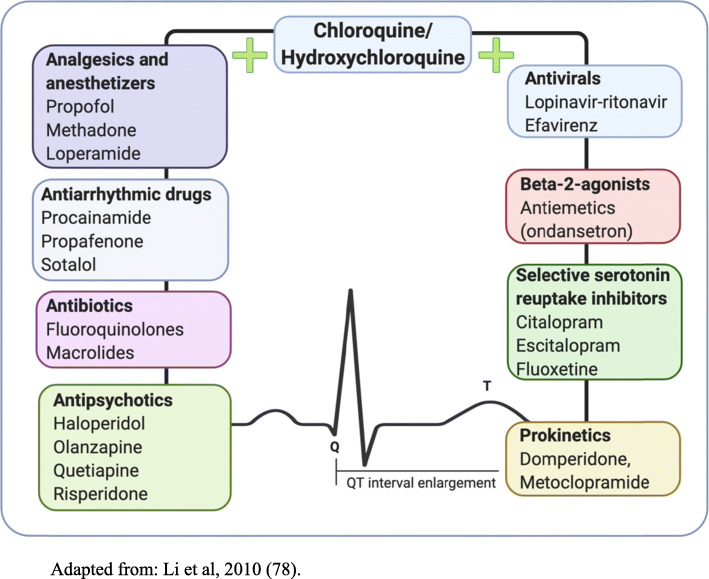
Main drug interactions with antimalarials related to QT interval enlargement. Adapted from: Li et al., 2010 [78]

Special attention should be given to other concomitant drugs, such as macrolides, quinolones, some antivirals and antipsychotics, because they can also lead to QT interval enlargement (Fig. 4) [13, 81].

## Recommendations

Since this is a narrative review, it is not possible to make formal recommendations, but suggestions for monitoring and proposal of key messages are valuable, and provide information about AM use for health-care providers, especially rheumatologists. These key messages are depicted in Table 2.

**Table 2:** Key messages regarding safety of treatment with antimalarial drugs

- Daily dose not greater than 5 mg/Kg - Regular screening for retinal toxicity according to risk factors - Monitoring of complete blood count at the beginning and during prolonged therapy - Physical examination with attention to muscle strength and reflexes - Monitoring of QT interval prolongation in at-risk patients - Caution in hepatic and renal impairment, use of other medications that lead to prolongation of the QT interval or that increase the serum level of antimalarials, alcoholism, concurrent antidiabetic agents, porphyria, psoriasis.	

## Conclusions

Given its multiple benefits, the use of AMs, preferably HCQ, should be encouraged to SLE patients, unless there is any contraindication. In other diseases like RA, pSS, APS, dermatomyositis and sarcoidosis some studies also show positive data, especially under specific circumstances. The majority of the side effects occur after a wide range of cumulative dosages.

It is a low-cost and widely available medication, whose safety profile is well known and acceptable. In addition, considering its pharmacokinetic properties (long half-life), it is possible to measure its serum concentration as a marker of treatment adherence and potential long-term toxicity, when necessary and available.

## Data Availability

Not applicable.

## Abbreviations

ACR: American College of Rheumatology
APS: Antiphospholipid syndrome
AM: Antimalarials
CQ: Chloroquine
CIMID: Chronic immune-mediated inflammatory rheumatic diseases
DMARD: Disease-modifying antirheumatic drugs
EULAR: European League Against Rheumatism
GLADEL: Grupo Latino Americano para Estudo do Lupus
HCQ: Hydroxychloroquine
HOMA: Homeostatic Model Assessment
pSS: Primary Sjögren syndrome
SLE: Systemic lupus erythematosus
RA: Rheumatoid arthritis
SBR: Brazilian Society of Rheumatology
